# Pathophysiology, Classification and Comorbidities after Traumatic Spinal Cord Injury

**DOI:** 10.3390/jpm12071126

**Published:** 2022-07-11

**Authors:** James Guest, Nilanjana Datta, George Jimsheleishvili, David R. Gater

**Affiliations:** 1 The Miami Project to Cure Paralysis, University of Miami Miller School of Medicine, Miami, FL 33136, USA; nxd693@med.miami.edu (N.D.); gxj150@med.miami.edu (G.J.); dgater@miami.edu (D.R.G.J.); 2 Department of Neurological Surgery, University of Miami Miller School of Medicine, Miami, FL 33136, USA; 3 Department of Physical Medicine and Rehabilitation, University of Miami Miller School of Medicine, Miami, FL 33136, USA

**Keywords:** spinal cord injury, tetraplegia, paraplegia, autonomic dysfunction, regeneration, comorbidities

## Abstract

The spinal cord is a conduit within the central nervous system (CNS) that provides ongoing communication between the brain and the rest of the body, conveying complex sensory and motor information necessary for safety, movement, reflexes, and optimization of autonomic function. After a spinal cord injury (SCI), supraspinal influences on the spinal segmental control system and autonomic nervous system (ANS) are disrupted, leading to spastic paralysis, pain and dysesthesia, sympathetic blunting and parasympathetic dominance resulting in cardiac dysrhythmias, systemic hypotension, bronchoconstriction, copious respiratory secretions and uncontrolled bowel, bladder, and sexual dysfunction. This article outlines the pathophysiology of traumatic SCI, current and emerging methods of classification, and its influence on sensory/motor function, and introduces the probable comorbidities associated with SCI that will be discussed in more detail in the accompanying manuscripts of this special issue.

## 1. Introduction

Spinal cord injury (SCI) is a life-changing neurological condition for which restorative treatments are yet to be achieved. SCI results from trauma, inflammatory diseases, tumors, vascular malformations, and increasingly degenerative cervical myelopathy [1]. Across etiologies, the pathomechanisms of injury and consequences to function have similarities. Fundamental injury mechanisms such as oxidative injury are similar to neurodegenerative diseases affecting the spinal cord such as amyotrophic lateral sclerosis [2]. This manuscript focuses on traumatic SCI due to the breadth of research on pathophysiology and the extensive development of classification systems. Due to traumatic SCI having a singular initial injury event, the temporal course of pathophysiological mechanisms, recovery, and evolution of resulting comorbidities is more stereotyped than other forms of SCI caused by inflammatory etiologies such as multiple sclerosis or viral myelitis.

Across the span of human history, SCI was mainly a fatal condition. Until the 20th century, SCI was not compatible with long-term survival, and a poor prognosis was described in historical medical writings [3]. Mortality was due to acute complications such as respiratory failure, subacute complications such as sepsis from pressure injuries or urinary tract infections, and chronic complications such as renal failure. During World War 2, the disciplines of SCI medicine advanced significantly through the prevention of urosepsis, pressure injuries, and the advent of rehabilitation [4]. Once survival after SCI was achieved, efforts to mitigate and repair the injury increased in importance. Further, a need for an international language of classification was evident.

We summarize the pathophysiology and classification of traumatic spinal cord injury (SCI) from the acute into chronic phases. The pathophysiology of SCI is a complex topic that could occupy an entire textbook with aspects unique to the nervous system’s organization and structure. Insights into pathophysiological mechanisms that follow SCI are essential for designing targeted therapeutic strategies and optimizing critical care practices. In this manuscript, we acquaint the reader with a current summary of accepted concepts and emerging scientific evidence that has recently expanded to include a systems-level understanding of the multi-organ consequences of SCI. We will discuss SCI classification and pathophysiology, as they are now being bridged through computational omics data analyses and biomarker studies including advanced neuroimaging. Finally, the comorbidities associated with acute and chronic traumatic SCI will be introduced.

## 2. Structural Disruption

The spinal cord white matter is exquisitely organized into longitudinal neuron cell processes in tracts, many of which are surrounded by myelin produced by oligodendrocytes. Motor and descending tracts from the brain include pyramidal (lateral and anterior corticospinal tracts) and extrapyramidal (rubrospinal, reticulospinal, olivospinal and vestibulospinal tracts) pathways. Pyramidal tracts from the cerebral cortex allow for voluntary control of the muscles of the body and face, whereas extrapyramidal tracts originate in the brainstem and provide unconscious, reflexive, or responsive control of musculature, i.e., muscle tone, balance, posture, and locomotion. Sensory and ascending pathways include the anterolateral, spinocerebellar, and dorsal columns that convey pain/temperature, unconscious proprioception (e.g., stretch), and position/vibration sensations, respectively. Gray matter contains the neuron cell bodies that receive and integrate signals and execute conscious and unconscious functions, and is segmentally organized for incoming sensory and outgoing motor axons. Of note, autonomic nervous system (ANS) preganglionic sympathetic cell bodies lie within the intermediolateral horns of the thoracolumbar spinal cord, whereas parasympathetic preganglionic cell bodies originate in the sacral segments of the spinal cord [5]. This organization is less strictly segmental with numerous interconnections between the paraspinal autonomic ganglia. Spinal cord neurons have critical interactions with astro- and microglia and around blood vessels where the blood-brain-barrier (BBB) is formed. The neuroglial extracellular space contains small quantities of extracellular matrix (ECM) glycoproteins and proteoglycans that anchor cellular cytoskeletons and stabilize synapses [6,7]. A significant consequence of SCI is disruption of this structure followed by scarring and infiltration of fibrotic cells that generate a permanently disorganized ECM [8].

## 3. Primary and Secondary Injury to the Spinal Cord

Historically, acute SCI pathophysiology has been classified into the primary instantaneous injury and secondary locally propagating progressive injury processes. It is not possible to modify the primary injury, but secondary damage can be attenuated or exacerbated in various ways. For example, post-SCI spinal cord compression can exacerbate the primary injury through ischemia [9]. Decompression can restore spinal cord flow, possibly reducing ischemic tissue death [10].

### 3.1. Primary Traumatic Injury

The initial mechanical forces traumatically imparted to the spinal cord-the primary injury-are the focal point from which secondary injury mechanisms extend. The primary injury can cause contusion, maceration, and laceration of the spinal cord, nerve roots, blood vessels, and dura [11]. Axonal disruption may be instantaneous [12]. Whereas the spinal cord usually is subject to some cyclic loading, tensile forces, and changing velocities of CSF flow, these do not exceed the tolerance of the tissue and cause any apparent injury. The spinal cord is biomechanically complex concerning anisotropy and interfaces such as the pial boundary, nerve root interfaces, restraining dentate ligaments, and vasculature. Uniquely, the spinal cord is suspended in a constantly moving fluid column of cerebrospinal fluid. A primary injury, by definition, exceeds the tolerance of these tissues to regular motions [13]. In acute traumatic SCI, this involves rapid spinal cord distortion by displacement of bone, intervertebral discs, and ligaments that concuss, stretch, or lacerate the tissues resulting in immediate damage to axonal processes and associated myelin, small blood vessels, astrocytes, microglia, and interruption of critical boundaries such as the BBB and pia mater. In SCI models, adequate contusion velocity is a crucial parameter to replicate actual rates of human injury [14]. One important example to illustrate the vulnerability of neural interfaces to primary injury is the avulsion of rootlets from the spinal cord caused by brachial or lumbosacral plexus stretch injuries [15].

The forces applied to the spinal column and spinal cord vary by injury type. In vehicular impacts that lead to survivable SCI, the dissipated energy is generally less than 200,000 joules with a velocity change of 47 km per hour and influenced by airbag deployment and seatbelts [16]. Extensive crash-test data has shown that energy dissipated in the impact varies exponentially with deceleration velocity. However, increasingly, non-fracturing impacts with much lesser injury forces occur in older adults with pre-existing cervical spinal stenosis [17]. While these do not lead to fracture fragment compression, the spinal cord may still be severely compressed [18]. Laceration and transection injuries occur through penetrating missile and knife injuries or sharp bone fragments. Missile injuries deliver focal damage but may increase injury volume due to terminal velocity-induced stretch cavitation [19]. Military penetrating injuries causing spine damage in recent blast wounds have been associated with a concomitant high rate of traumatic brain injuries [20]. Cervical fractures can also lead to occlusion and dissections of the nearby vertebral arteries contributing to ischemia [21].

### 3.2. Secondary Injury

Neurons are especially vulnerable to injury because they have a large volume compared to other cells and numerous membrane specializations, including synapses and complex interactions with astrocytes and myelin-forming oligodendroglia. While representing only 10% of the spinal cord structure, neurons have as much as 99.7 percent of their cytoplasm in axonal and dendritic processes with a relatively small cell body and nucleus volume resulting in complex spatial metabolic and transport needs [22]. Further, neurons uniquely transmit action potentials via rapid ionic currents through several types of membrane channels. After SCI, these currents are dysregulated, leading to severe cell stresses such as excessive calcium influx [23] that can damage mitochondria and other cell structures [24].

The discipline of SCI neuroprotection evolved as it was increasingly understood that the injured spinal cord has limited inherent repair capability [25]. Thus, protection of at-risk tissue is of paramount importance, as it is in stroke. We now understand that clinical interventions, including rapid spinal cord decompression [26] and pharmacologic stabilization of neurogenic shock [27], are neuroprotective. The logistical intensity of acute SCI critical care has facilitated several clinical trials of neuroprotection [28]. The first attempts at neuroprotection were based on hypothermia and continue to this day [29,30,31,32,33,34].

A multitude of secondary consequences follow SCI. Several such as lipid peroxidation [35] and excitoxicity [36] have been targeted by clinically tested therapeutics. One meaningful way to consider SCI mechanisms is along the axis of time. Secondary injury begins within seconds following the initial insult and peaks within days of injury [37], but the consequences continue indefinitely [38]. The major pathophysiologic events differ by temporal stage (acute-subacute-chronic).

Vascular damage is an immediate consequence of SCI that is followed by ischemia. Local ischemia results from primary disruption of vessels, vasospasm, and intravascular thrombosis. The highly vascularized grey matter that contains neurons is susceptible to injury with a fivefold higher density of capillary beds reflecting its higher metabolic demands. Traumatic disruption of grey matter with the progression of the injury volume and extravasated blood is notable in severe injuries [39] and has been measured with MRI [39] and ultrasound [40]. Activation of the endothelial sulfonylurea channel 1 has been linked to progressive capillary fragmentation after SCI [41].

After severe SCI, acute loss of descending inputs to sympathetic preganglionic neurons causes neurogenic shock, characterized by reduced peripheral vascular resistance and relative dominance of vagal tone with bradycardia and depressed myocardial function. Shock can exacerbate ischemia at the spinal cord injury site and affect other parts of the neuroaxis and critical organs such as the kidneys. Ischemia may be followed by periods of reperfusion, which may exacerbate neuronal cell death by producing free radicals and toxic byproducts, worsening oxidative stress and cell damage [42]. The optimal level of post-injury systemic blood pressure support has been recently complicated by the finding that upper limits are critical to avoid hemorrhage propagation [43].

Oxygen is essential to normal cellular energy production but can cause extensive cell damage and even cell death if a delicate balance of free radicals is exceeded. The hallmark of oxidative stress is the production of free radicals such as superoxide and nitric oxide. These electron imbalanced oxygen moieties have essential physiological roles in the vascular endothelium and immune cells at low transient concentrations. However, they are toxic at higher levels and contribute to neurologic disease. Free radicals’ primary pathological intracellular source is ischemic or hypoxic mitochondria, whose progressive damage leads to electron transport failure, cytochrome C release, and apoptosis-inducing factor (AIF), activating cell death [44]. A hallmark of oxidative stress is that it can be exponentially auto-propagating.

Bilayer lipid membranes are the critical boundaries of cells and organelles, including mitochondria, allowing life through regulated function. Membrane pores and channels enable exquisite control over critical processes such as cellular energetics, osmotic control of volume, and the transmission of electrical current. Homeostasis expends and renews ATP energy to regulate the concentrations of ions such as Na^+^, K^+^, Cl^−^, and Ca^2+^ across cell membranes as the basis for action potential transmission. Early insights into the pathophysiology of secondary SCI focused on glutamatergic excitoxicity [45] and membrane injury with associated abnormal ionic fluxes, generation of free radicals, and mitochondrial dysfunction [46]. These biochemical insights led to an interest in applying steroids to acute SCI and several other targets [47].

Lipid peroxidation, a propagative peroxyl radical chain reaction, starts when reactive oxygen species interact with polyunsaturated fatty acids in the cell membrane [48]. It is especially damaging because the chain reaction can continue until no more unsaturated lipids are available. The final products of the lipid peroxidation are 4-hydroxynonenal (HNE) [49] and 2-propenal, which is highly toxic to the stressed cells by causing DNA and mitochondrial injury, activating caspases, and p38MAPKinase [50].

“Excitotoxicity” was one of the first neural pathomechanisms described and is relevant in multiple neurological diseases [45]. It is a paradigm of how critical neural processes such as synaptic transmission may become pathological when energy homeostasis is lost. Glutamate is the major excitatory neurotransmitter of the CNS. It binds to two classes of receptors: ionotropic and metabotropic, present on the surface of neurons, glial cells, and endothelial cells. Glutamate is found in all physiological fluids at varying levels and is tightly controlled in the synaptic cleft through astrocyte uptake transporters and conversion to glutamine [51]. In excitotoxicity, excessive glutamate starts a cascade of neurotoxicity that ultimately leads to the loss of neuronal function and cell death [52].

Immediately after SCI, there is a significant rise in extracellular potassium and glutamate [53] that activates AMPA-kainate ionotropic receptors allowing gradient-driven sodium influx inside the cells [54]. At negative membrane potentials, magnesium blocks the ionotropic NMDA receptor channel; however, when neurons are progressively depolarized, magnesium is extruded, and calcium enters the cell. During ischemia and hypoxia, perturbed cellular energy states impair restorative sodium, potassium, hydrogen, and calcium transport, further potentiating glutamate release from neurons. Astrocytes also lose control of their excitotoxic amino acid transporters.

Mammalian life requires oxidative metabolism mediated through mitochondria. Membrane-bound mitochondria closely control hydrogen ion levels to use oxygen in biochemical reactions. The electrochemical proton gradient allows the generation of ATP from ADP, the most critical cellular energy source. Dephosphorylation of ATP releases energy, as an example, causing the Na channel protein conformational shift that extrudes sodium from the cells at the surface membrane to repolarize neurons. Calcium enters the mitochondria through the mitochondrial calcium uniporter (MCU) to regulate oxidative phosphorylation and increase ATP production [55]. Mitochondria can transiently buffer a high calcium concentration following intense stimulation by opening the mitochondrial permeability transition pore (MPTP) to reduce calcium levels. However, when calcium levels and oxidative stress are excessive, MPTP opening leads to mitochondrial swelling, loss of effective electron transport, and the release of cytochrome C, triggering cell death [46]. Mitochondrial calcium overload also activates NADPH oxidate (NOX), inducing the generation of superoxide and nitric oxide by the electron transport chain. Severe cellular stress and high mitochondrial calcium activate the calcium-sensitive protease calpain. Calpain cleaves mitochondrial apoptosis-inducing factor (AIF), which translocates to the nucleus and cleaves DNA, further accelerating cell death [56]. Within the cytosol, activated calpain degrades essential proteins of the cytoskeleton and critical sodium-calcium exchangers such as NCX3 [57].

An emerging class of therapeutics inhibits the DNA damage sensor and signaling enzyme poly(ADP-ribose) polymerase(PARP). PARP1 repairs damaged mitochondrial DNA. Severe cell stress can lead to hyperactivation of PARP, causing severe depletion of the essential energy substrates NAD and ATP. Then, even glycolytic energy metabolism fails, activating “cell suicide” [58].

SCI may take months to resolve and typically evolves with tissue loss at the point of maximum injury. Cell death leads to depletion of essential neurons, glia, and vasculature. Although some cells are instantly destroyed, cell death is a protracted process that lasts for days to weeks. Defined cell death mechanisms include necrosis, necroptosis, apoptosis, and autophagy. Oligodendroglia are highly susceptible to apoptotic cell death resulting in axonal demyelination [59]. Necroptosis is induced by the inflammatory cytokine TNF-alpha binding to TNF receptor 1 (TNFR1).

**Figure 1:**
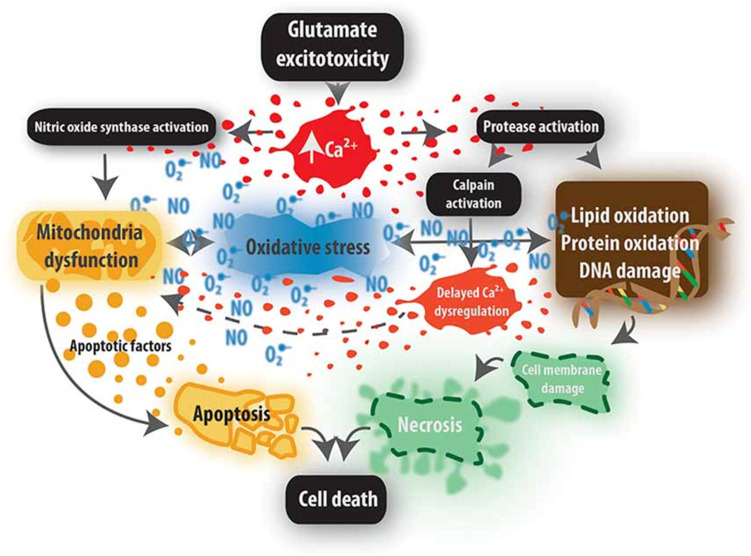
The Figure illustrates the pathophysiological events that accompany excitotoxicity including mitochondrial stress, elevated Ca^2+^ with associated enzyme activation and cell death. Reprinted from Ref. [69].

After discoveries of secondary biochemical injury mechanisms, a subsequent generation of researchers brought attention to the adverse effects of inflammation, focusing on damage caused by cytokines, inflammasome complexes [60], neutrophils [39], and subsequently macrophages [61]. Early in-migration of neutrophils via CD11d/CS18 integrin-binding releases several tissue destructive factors [62]. Human studies confirmed an early injury response by local microglia and neutrophils followed by blood-borne macrophages [39]. Experimental depletion of hematogenous macrophages reduced the magnitude of secondary tissue loss [63]. The focal injury leads to a reactive glial boundary formation with extracellular matrix deposition and fibrosis [64]. A new controversy in SCI research has suggested that long-held views about glial scarring after SCI should be reinterpreted as a critical regulator of the spread of the focal post-SCI inflammatory response [65]. Matrix metalloprotease enzymes have emerged as essential regulators of the extracellular environment after SCI contributing to both injury and repair [66]. Macrophages have also been shown to have damaging inflammatory and tissue reparative activities after SCI [67]. The inflammatory response activates gene transcription through key regulatory cytokines such as NF kappa B [68]. Figure 1 Request permission to reproduce from [69].

Concepts continue to evolve and now include events remote to the injury site itself are identified that regulate the extent of injury site neutrophil recruitment [70]. In the last decade, systemic events such as pneumonia have been implicated to amplify secondary injury [71]. Changes in the gut’s microbiome may also contribute to secondary injury [72]. An added layer of systemic complexity is the evolving understanding of the immune system, including discovering the post-SCI immune deficiency syndrome [73] whereas there is potential induced autoimmunity due to exposure of SCI exposed antigens such as myelin basic protein [74,75].

Treatment of secondary injury. The concept that secondary damage can be diminished has provided the rationale for numerous clinical trials of agents that directly address known mechanisms. These have included steroids, NMDA receptor antagonists [76], Riluzole [77], and magnesium [78]. Early surgical decompression in the first 24 h post-injury appears to have a neuroprotective benefit [28].

## 4. Bioinformatics and Acute Sci Pathophysiology

In the past, there was a tendency for pathophysiological thinking to be compartmentalized into distinct mechanisms such as “ischemia” and “excitotoxicity.” We now increasingly realize from bioinformatics data analysis that there are no singular molecular perturbations. One change begets numerous others, as indicated in animal model data [79,80]. There is also interest in bridging pathophysiology and classification by way of injury biomarkers, including damaged structural molecules such as GFAP and neurofilament and signaling molecules such as miRNAs and exosomes [81].

## 5. Classification of Traumatic Sci

Clinical classification methods should be able to be implemented broadly without excessive cost. SCI classification tools serve three primary purposes: (1) to both accurately describe individual injuries and assign categories; (2) inform prognosis; (3) serve as recovery outcome measures over time. More recently individuated molecular pathobiology is being explored of which one example is to analyze the RNA content of peripheral white blood cells [82]. Classification tools need to define distinct categories and measurable quantities to be valid. Injuries vary by the level of the spinal cord affected, the injury severity, and whether the injury involves or is below the conus medullaris and thus distal to motor neurons. Classification is also essential to detect deterioration in neurological function that can occur in the acute or chronic phases. The main current classification methods are clinical, MRI-based, and molecular. Molecular methods need a frame of reference and thus are currently interpreted through clinical grading.

### 5.1. Clinical Classification

The most widespread SCI assessment and classification methods are the International Standards for Neurological Classification of Spinal Cord Injury (ISNCSCI), developed by the American Spinal Injury Association (ASIA) in 1982 [83]. These evolved from the Frankel Classification, which defined five ordinally ranked grades (A to E) [84]. Significant differences between the earlier Frankel Classification and ISNCSCI are the specification of level of injury, the inclusion of the prognostically important anorectal exam, and the designation of key sensory points and muscles. Injury incompleteness is primarily defined by sacral sparing [85], which has repeatedly demonstrated prognostic value and has an anatomic basis related to spinal tract lamination [84,85]. The ISNCSCI standards were endorsed by the International Spinal Cord Society in 1992. They underwent revisions, most recently in 2011 [86] and 2019 [87], as specific limitations became apparent based on overall experience (https://asia-spinalinjury.org/international-standards-neurological-classification-sci-isncsci-worksheet/, accessed on 15 May 2022). ISNCSCI provides a low-cost international methodology comprising a sensory, motor, and sacral segment exam to formulate the impairment grade (AIS), neurological level of injury (NLI) and quantify the extent of residual motor and sensory function. For the sensory exam, the 28 dermatomes for each body side are tested using a cotton swab for light touch and a pin to detect sharpness (dorsal column and spinothalamic sensory systems, respectively). The motor exam tests strength in five key muscles of each extremity, and the anorectal examination assesses deep anal pressure (DAP) and voluntary anal contraction (VAC). Scores are ordinally ranked; sensory scores range from 0 to 2 (0 for absent sensation, 1 for altered sensation, and 2 for normal sensation). Motor scores range from 0 to 5 (0 representing no detected motor function, 1—trace contraction, 2—a full range of movement in gravity eliminated state, 3—a full range of motion against gravity but not against applied resistance, 4—a full range of movement against slight resistance, and 5—normal strength). The neurological level of injury (NLI) is defined as the last fully intact segment on both sides of the body.

The ASIA Impairment Scale (AIS) defines the degree of impairment based on the sacral segment exam and motor scores below the injury level. Its relationship to recovery extent is well-validated [88]. There are important differences between the AIS and motor and sensory scores. AIS is categorical and linked to the preservation of spinal cord tracts. Motor scores are semi-quantitative and linked to spinal cord segmentation. Scale definitions are:

- A = Complete. No sensory or motor function is preserved in the sacral segments S4–S5.
- B = Sensory incomplete. Sensory but not motor function is preserved below the neurological level. It includes the sacral segments S4–S5, and no motor function is preserved more than three levels below the motor level on either side of the body.
- C = Motor incomplete. Motor function is preserved below the neurological level, with detectable voluntary rectal contraction and/or more than half of key muscles below the single neurological level of injury have a muscle grade < 3/5 (Grades 0–2).
- D = Motor incomplete. Motor function is preserved below the neurological level, and at least half of key muscles below the NLI have a muscle grade > 3.
- E = Normal. If sensation and motor function as tested with the ISNCSCI are graded as normal in all segments, and the patient had prior deficits, the AIS grade is E. Note, pathological spasticity and autonomic dysreflexia may be present despite normal motor and sensory function. Someone without an SCI does not receive an AIS grade.

Initial autonomic assessment should include the International Standards to determine remaining Autonomic Function after Spinal Cord injury (ISAFSCI) [89] used in concert with the International Standards for the Neurological Classification of SCI (ISNCSCI) [88]. This tool documents cardiovascular, bronchopulmonary and sudomotor symptomatology as well as genitourinary, bowel and sexual function providing a clinical “score” of autonomic dysfunction [89]. Additional objective testing might include non-invasive sympathetic skin sweat responses (SSR) to electrophysiologic stimulation of median and tibial nerves to quantify risk of autonomic dysreflexia. In a study by Curt et al., SSR significantly corresponded to the neurologic level injury by ISNCSCI exam in complete SCI, and predicted the likelihood of developing chronic autonomic dysreflexia, however SSR did not show significant predictive value in those patients with incomplete lesions [90].

Specific issues can compromise the accurate performance of the ISNCSCI, especially in the earliest injury phases when the information may be essential for therapeutics clinical trial enrollment. Intubation or tracheostomy hinders the necessary reciprocal communication between the tester and examinee. The anorectal exam may not be feasible when patients are in ICU, or cannot be turned to the side. Alternative techniques have been suggested as a substitute for the anorectal exam, including S3 pressure assessment [91], and S1 sensory tests [92], but they have not been thoroughly field-tested. Another limitation to accomplishing an accurate complete exam is lack of time, especially if the exam needs to be obtained before surgery, so an abbreviated exam has been developed and suggested as an alternative [93]. ISNCSCI does not test daily life functions and independence for which the Spinal Cord Independence Measure (SCIM) has been developed [94]. There is a continuing need for alternate classification methods for those who cannot be examined due to coma, polytrauma, or traumatic brain injury (TBI).

Is the ISNCSCI a good outcome measure for longitudinal research? Research studies require validated outcome measurements. Changes in ISNCSCI parameters such as motor score and AIS have been extensively utilized in research as outcome measures [95,96]. The sensory exam is less well-validated than the motor component [97]. However, the ISNCSCI does not test actual functions such as hand grasping and walking that are composites of multiple elements of sensorimotor control. Other outcome measures developed to assess these functions include the Graded Redefined Assessment of Strength, Sensation and Prehension (GRASSP) [98] for the upper extremity and the Walking Index for Spinal Cord Injury (WISCI/WISCI II) for assisted walking [99]. A composite of the ISNCSCI motor score and the SCIM, version 3 (SCIM-III), the Spinal Cord Ability Ruler (SCAR) has been proposed to linearize the assessments and create a measure of neurological and daily life function [100]. ISNCSCI scores have been combined with other outcomes in algorithms that increase their predictive power for e.g., independent ambulation [101]. In cervical segments, their contiguous values spanning the injury region, known as the zone of partial preservation, predict recovery in segments of high clinical recovery value [102]. As an outcome measure, motor scores and SCIM-III scores have been combined to create a linearized measure that is being tested in clinical studies [100]. Another emerging classification tool is to apply recursive partitioning to create inference trees that stratify the individual using ISNCSCI or other data into prognostic groups [103].

### 5.2. MRI Classification

Most patients with SCI undergo MRI scanning soon after injury, and MRI findings have consistently been linked to the recovery prognosis. MRI is very sensitive to changes in tissue and can define compression severity with the linear extent of edema and hemorrhage linearly correlated to both SCI severity and recovery [104]. Changes between MRI exams are also predictors, with a greater rate of lesion expansion measured across two sequential acute MRIs linked to less recovery [105]. MRI can also show the adequacy of surgical decompression, which correlates to outcome [106]. However, despite the great value of MRI, standardized quantitative measures have not been widely validated in large studies. One measure of interest is the Brain and Spinal Injury Center score (BASIC Score) assigns axial T2-weighted MRI scans with ordinal values from 0 to 4 [107]. High reliability between BASIC scores and AIS grades was found with the potential to distinguish between AIS grade A patients who may have conversion or not, considered important for therapeutics clinical trials. BASIC, limited to a single axial measure of intrinsic spinal cord signal abnormality, may have greater predictive power when combined with measures of extrinsic cord compression and lesion length [108,109].

- BASIC 0: No appreciable intramedullary cord signal abnormality.
- BASIC 1: Intramedullary T2 hyperintensity confined to central gray matter.
- BASIC 2: Intramedullary T2 hyperintensity extends beyond the expected gray matter margin to involve spinal white matter but does not involve the entire transverse extent of the spinal cord.
- BASIC 3: Intramedullary T2 hyperintensity involves the entire transverse extent of the spinal cord.
- BASIC 4: Grade 3 injury plus discrete T2 hypointense foci, consistent with macro-hemorrhage.

Other MRI biomarkers that provide injury severity and prognostic microstructural information are diffusion tensor imaging [110] and axial diffusivity with correlations to both ISNSCI motor scores and SCIM-III at one-year post-injury [111]. At later time points, the width of mid-sagittal image tissue bridges is correlated with the locomotor outcome as measured by the six-minute walk test [112]. Regarding chronic injury, emerging data indicate that SCI is associated with chronic neurodegeneration, as revealed by increased brain ventricular volumes after SCI [113]. MRI measures can also identify changes in myelination through bound water fractions [114], an important contributor to post- SCI pathology [115].

### 5.3. Molecular Classification

Neither clinical nor MRI biomarkers provide molecular-cascade level temporal resolution. Molecular classification could allow a greater mechanistic linkage to the evolving injury to define therapeutic targets and effects. Substantial progress has been made in chemical biomarker research to inform both pathophysiology and classification. Biomarkers are biological compounds derived from accessible body fluids that correlate to pathophysiology, damaged structures, and signaling cascades. For SCI, these include structural proteins such as neurofilament [116] and tau [117] that correlate with injury severity and prognosis. Injury causes cell disruption releasing cellular fibrils such as neurofilament and GFAP that may be found in the cerebrospinal fluid and at lower concentrations in serum [118]. Their measured quantity has been linked to spinal cord tissue injury magnitude. Kwon and colleagues reported that a combination of CSF tau, GFAP, and S100β from 24-h CSF samples predicted AIS conversion at six months more accurately than MRI or inflammatory biomarkers [119]. In subsequent studies, these investigators identified a microRNA from acute CSF samples, 24 h miR-423-3p, that identified those patients with acute AIS A that converted their AIS grade at six months [120]. In a metabolome analysis, CSF levels of citrulline, glycerol, and N-methyl-D-aspartic acid correlated with presenting AIS grade [121]. It has also been discovered that readily available blood test markers, such as serum albumin, may also be prognostic markers [122]. Neurophysiological measures are also accessible biomarkers that may correlate in a sensitive manner with neuroplasticity and possibly provide an early indication of therapeutic effects [123].

## 6. Prognosis for Recovery

Classification is essential to creating treatment and hospital discharge plans. A combination of the ISNCSCI and the (GRASSP) scores has demonstrated predictive value for clinical decision-making for rehabilitation interventions and clinical trial inclusion when incorporated into conditional inference algorithms [124]. The AIS has consistently demonstrated predictive significance with significantly fewer people with an initial diagnosis of AIS grade A than those with AIS grade B converting to incomplete motor status at follow-up. About 50% of those with initial AIS grade B convert to incomplete motor status with higher rates of lower extremity motor score gains and sensory score changes [88].

Methods for stratification of acutely injured persons are critical in designing clinical trials. In early phase studies, the ability to establish a therapeutic proof of concept is essential to development. At this clinical trial phase, it is appropriate to emphasize enrolling subjects whose injury profiles are more likely to show an effect [125,126].

Systemic response markers from the blood also have predictive value. In an analysis of data from the Sygen study [126], higher serum albumin levels between 1–4 weeks post-injury were associated with higher motor scores at one year [31]. Recently a high initial neutrophil-lymphocyte ratio has been found to portend less neurological recovery [127].

Bioinformatics, Machine Learning and Acute SCI Pathophysiology

The biomarkers above can classify injury severity, but it would be valuable to individualize classification and link it more specifically to the evolving pathophysiology, especially that which can be targeted therapeutically or indicates harmful intercurrent events. Bioinformatics use computational analysis of omics data such as gene transcription and protein expression to find linkages between molecular cascades. If samples are timely, this could enable a temporal association between gene transcription and biochemical pathways [80]. Integrated systems analysis can allow comparison of large preclinical research datasets to human data to detect conserved gene expression networks [128] and similar relevant pathophysiology [129]. Thus, bioinformatics classification may increase the resolution of biomarkers. Machine learning interrogation of preclinical and human datasets can disclose unappreciated clustering and identify important novel targets [130].

## 7. SCI Comorbidities

### 7.1. Functional Mobility and Activities of Daily Living (ADLs)

Motor paralysis is a hallmark feature of SCI, with level of injury (LOI) and completeness of injury (AIS) serving as critical metrics for functional outcomes. For each level of SCI, functional outcomes have been established to assist with determining the equipment and techniques required to optimize independence [131]. Those with persisting C1–C3 AIS A-C tetraplegia will likely require 24-h attendant care, mechanical ventilation and/or diaphragmatic pacing for respiratory function, and will require total assistance with transfers, bed mobility, feeding, grooming, hygiene, dressing, bathing, and toileting, but should be able to use a power wheelchair for household and community mobility, as well as providing pressure relief using power wheelchair features of tilt and recline. Those with C4 AIS A-C tetraplegia will have similar abilities, but most likely will not require ongoing mechanical ventilation. With active elbow flexion, persons with C5 AIS A-C tetraplegia may be able to feed themselves and provide some assistance with grooming if provided appropriate adaptive devices; driving with hand controls becomes possible, and attendant care hours may be somewhat reduced (16–24 h/day) depending upon the individual’s body habitus and other comorbidities. Those with C6 AIS A-C will be able to use wrist extensors to facilitate grasp, gaining additional abilities with dressing, bathing and perhaps bowel and bladder management. With active elbow extension, the person with C7 or C8 AIS A-C tetraplegia is more likely to perform modified independent transfers, pressure relief and manually propel an ultralightweight wheelchair; personal care and homecare assistance hours may drop to 8 h/day. T1-T9 AIS A-C paraplegia should be modified independent for ultralightweight wheelchair propulsion, pressure relief, bladder/bowel management and ADLs, although truncal balance and abdominal activation for cough will still be compromised. Those with T10-L1 AIS A-C paraplegia will have improved truncal balance and should be able to generate a significant cough to clear secretions; some will be able to ambulate with bilateral knee-ankle-foot orthoses (KAFOs) and bilateral forearm crutches, but most will still need an ultralightweight wheelchair for community mobility. Persons with L2-S5 AIS A-C paraplegia will have similar function, but with additional voluntary hip flexion, knee extension and ankle function at lower levels of SCI. Persons with AIS D may have sufficient voluntary lower extremity function for ambulation with or without orthoses and assistive devices but may be limited for community distances because of spasticity and fatigue; an ultralightweight wheelchair should still be provided.

### 7.2. Respiratory Dysfunction

Persons with SCI above T8 will have neurogenic restrictive lung impairments due to intercostal and abdominal muscle paralysis. Not only will they be unable to generate sufficient cough for clearing secretions, but they will also have diaphragmatic flattening as abdominal contents fall down and out. An abdominal binder is essential to compensate for the abdominal muscle paralysis and improve breathing mechanics [132]. Because of sympathetic blunting, those with SCI above T6 will also have neurogenic obstructive lung impairments with parasympathetic dominance creating bronchoconstriction, hyperreactive airways and copious mucus secretions [133]. Sleep apnea is highly prevalent in SCI, whether the person has tetraplegia or paraplegia, and should be assessed as indicated [134].

### 7.3. Cardiovascular Dysfunction

As the sympathetic preganglionic cell bodies lie in the intermediolateral horns of the thoracolumbar spinal cord, SCI can result in significant sympathetic blunting for persons with SCI above T6. Neurogenic bradycardia is likely to occur and may require temporary pacing in the early days after injury. Neurogenic orthostatic hypotension (NOH) can be especially problematic during the early days/weeks of acute rehabilitation, and may require pharmacological as well as mechanical interventions [90,135]. The combination of venous stasis, trauma, and hypercoagulability after traumatic SCI contributes to high risk of venous thromboemboli, and prophylaxis is recommended during the first 8–12 weeks after injury [136]. The reduced afterload associated with NOH and diminished preload due to reduced muscle pump activity/venoconstriction both contribute to an adaptive myocardial atrophy [137,138,139]. In a seeming paradox, persons with SCI above T6 are also at high risk for autonomic dysreflexia (AD), a life-threatening hypertensive crisis that involves unabated sympathetic reflex activation of the splanchnic vascular bed in response to a noxious stimulus below the level of injury. The most likely stimulus of AD is bladder distension or infection, although bowel etiologies are also very common. Initial management is to sit the person upright, find and remove the source, and if necessary, provide acute pharmacological intervention to regain baseline blood pressure [140].

### 7.4. Cardiometabolic Syndrome

The combination of sympathetic blunting, muscle paralysis/atrophy, bone loss and anabolic deficiency contributes to a markedly diminished basal metabolic rate that is seldom matched by concomitant reduction in dietary intake; the net result is neurogenic obesity [141,142]. The profound increase in adiposity contributes to the metabolic syndrome, including diabetes mellitus, dyslipidemia, and vascular inflammation, placing individuals at very high risk of coronary artery disease [143].

### 7.5. Neuropathic Pain

Disruption of the spinal cord interrupts primary and secondary pain afferents that are particularly susceptible to excitatory amino acids and neurotransmitters associated with the SCI [144]. The perception of burning, tingling dysesthesias can be highly distracting and distressing to the individual during acute rehabilitation and in the chronic care setting, and may be experienced by as many as 60–80% of persons with traumatic SCI [144,145,146,147].

### 7.6. Spasticity

Spasticity has been commonly defined as a velocity dependent increase in muscle tone due to exaggerated stretch reflexes [148], or disordered sensorimotor control, resulting from an upper motor neuron lesion, presenting as intermittent or sustained involuntary activation of muscles [149]. It results from disinhibition of supraspinal pathways that typically dampen excitatory post synaptic potentials in the reflex final common pathway in the neurologically intact individual. While spasticity can be used to facilitate mobility and functional activities, it can also result in painful and problematic involuntary muscle spasms and contribute to muscle contractures [150].

### 7.7. Neurogenic Bladder

Suprasacral SCI will result in uncoordinated and pathologic bladder spasticity and sphincter spasticity referred to as detrusor—sphincter dyssynergia. The subsequent pressures within the bladder can exceed that required to maintain ureteral closure, with a high likelihood of vesicoureteral reflux and subsequent renal compromise. Neurogenic bladder needs to be carefully assessed [151] and managed in order to prevent upper tract deterioration, AD, and to prevent urinary incontinence [152].

### 7.8. Neurogenic Bowel

As with the neurogenic bladder, suprasacral SCI will result in uncoordinated and pathological bowel and sphincter spasticity referred to as rectal—sphincter dyssynergia. Additional comorbidities will likely include the development of constipation, hemorrhoids, and bowel incontinence, and recent guidelines have been provided to minimize these concerns and optimize quality of life [153].

### 7.9. Pressure Injuries

Unfortunately, persons with SCI are at high risk of developing pressure injuries due to lack of sensation, immobility, or both. Recent guidelines have been updated to emphasize pressure injury prevention, but also to optimize assessment and treatment interventions [154].

### 7.10. Bone Metabolism Dysfunction

Early in SCI, skeletal bones below the level of injury are subject to osteoclastic > osteoblastic activity due to mechanical unloading, anabolic deficiency, sympathetic dysfunction, and systemic inflammation. Young men are particularly at risk for immobilization hypercalcemia within the first weeks after SCI. Heterotopic ossification is likely to occur in the hip and knee joints, probably as a result of increasing spasticity that prompts the migration of mesenchymal bone cells [155]. As time progresses, lower extremity osteopenia is likely to occur in persons with both tetraplegia and paraplegia, although manual wheelchair propulsion may actually increase bone density at the upper extremities [156].

### 7.11. Sexual Dysfunction, Infertility and Pregnancy after SCI

Following SCI, self-efficacy, physical appearance, social acceptance, changes in roles and decline in sexual desire may impair an individual’s sexuality and desire for intimacy. The ability to experience the usual sexual response cycle (excitement, plateau, orgasm, and resolution) may be limited by sensory, motor and autonomic deficits depending upon the LOI and completeness of SCI. Whereas males often have infertility issues after SCI [157,158], most women will regain usual menstrual cycles within 3–6 months of SCI, and need to be made aware of special issues related to pregnancy, labor, delivery, and even nursing that can precipitate life-threatening AD [159,160].

### 7.12. Psychosocial Dysfunction

Following SCI, reactive depression is common, impacting relationships and family, finances, employment, living situation, and community reintegration. Premorbid psychological issues may be amplified, and coping, resiliency, mood disorders, self-harm behaviors, and sometimes concomitant brain injury can make SCI especially challenging from a mental health perspective [161].

### 7.13. Summary

The mechanisms of post-SCI injury propagation are increasingly understood, with some completely new elements being discovered. This likely means that other mechanisms are yet to found. Clinical classification methods such as the ISNCSCI have been well validated to classify injury severity, indicate prognosis, and measure recovery. MRI classification methods allow actual pathophysiological events such as edema and hemorrhage to be directly visualized, and their early appearance strongly correlates with injury severity and outcome. Biomarkers of injury measured from CSF and serum may correlate with the potential for therapeutic benefit of applied therapeutics, allowing calibration of therapeutic doses, durations, and determination of responders. Computational methods to integrate clinical and molecular data are informing prognosis and establishing the major triggers in injury cascades. Further, insights into systems biology provide for more integrated insight into the evolution of a person’s injury. It is sobering to realize the many comorbidities associated with SCI, and specialty care is essential to manage these complex diagnoses.

## Data Availability

Not applicable.
